# Tracing the Geographic Origin of the Pine Wilt Vector *Monochamus alternatus* Using Carbon Stable Isotope Analysis and Spatial Modeling

**DOI:** 10.3390/insects17050457

**Published:** 2026-04-27

**Authors:** Jun Ding, Zeshi Qin, Zhashenjiacan Bao, Juan Shi

**Affiliations:** 1 Sino-French Joint Laboratory for Invasive Forest Pests in Eurasia, Beijing Forestry University, Beijing 100083, China; 2 Beijing Key Laboratory for Forest Pest Control, Beijing Forestry University, Beijing 100083, China; 3 Hebei Xiong’an New Area City Ecosystem Observation and Research Station, Xiong’an 070001, China

**Keywords:** *Monochamus alternatus*, carbon stable isotope, geographical origin, traceability, co-Kriging interpolation

## Abstract

This study explored the application of carbon stable isotopes for tracing the geographical origin of *Monochamus alternatus*, an insect vector responsible for spreading pine wilt disease. The primary vector of pine wilt disease, an aggressive disease caused by the pine wood nematode and affecting pine forests, is *Monochamus alternatus*. Samples of *Monochamus alternatus* were collected from 12 provinces across China, and their carbon isotope ratios (δ^13^C) were measured. By analyzing the correlation between these ratios and various environmental factors, including latitude, longitude, altitude, and bioclimatic conditions, it was found that precipitation seasonality and solar radiation were the most important factors influencing the carbon isotope ratio of *Monochamus alternatus*. The spatial distribution of *Monochamus alternatus* carbon isotopes in China was predicted using the co-Kriging interpolation method, incorporating these two environmental variables. The findings revealed a gradient in the carbon isotope ratio of *Monochamus alternatus*, which could help differentiate the species across various geographical regions in China.

## Simple Summary

This study employed carbon stable isotope analysis to trace the geographic origins of *Monochamus alternatus*, the primary vector of pine wilt disease. Samples were collected from 12 provinces across China, and carbon isotope ratios (δ^13^C) were analyzed in relation to environmental factors. Precipitation seasonality and solar radiation were identified as the dominant factors influencing δ^13^C variational factors. It was found that precipitation seasonality and solar radiation strongly influence these ratios. Using a spatial modeling technique, a map of carbon isotope distribution across China was created, showing clear regional differences. This method can help identify where the insects come from, supporting efforts to control and prevent the spread of pine wilt disease.

## 1. Introduction

Pine wilt disease (PWD), also referred to as pine wilt, is a systemic infectious disease caused by the pine wood nematode *Bursaphelenchus xylophilus* [1]. It primarily affects *Pinus* species and a few non-Pinus conifers, such as *Larix olgensis*. Infected pine trees can succumb to the disease within 40 days. Statistics indicate that import and export restrictions due to pine wilt disease have resulted in annual losses of approximately USD 150 million in the United States and USD 700 million in Canada [2]. The most severe outbreak of the pine wilt disease is currently occurring in China. Over the past 40 years, the disease has progressively worsened in China. By 2021, it had affected 742 county-level administrative regions across 19 provinces (autonomous regions and municipalities directly governed by the central government), covering an area of 171.65 million hm^2^ [3].

Pine wilt disease spreads naturally through the movement of vector insects between infected and healthy trees. While several insect species can carry pine wood nematodes, it is predominantly certain species of *Monochamus* that are effective at transmitting the disease [4]. In East Asia, *Monochamus alternatus* is the primary insect vector, with adults carrying thousands to even hundreds of thousands of nematodes on the surface of their body. This insect is characterized by its supplementary feeding habits, high transmission efficiency, and significant harm. In the high latitudes of East Asia, the key vectors are *Monochamus saltuarius* and *Monochamus sutor* [5,6], while in Europe, the main insect vector is *Monochamus galloprovincialis* [7]. In North America, over a dozen species of pine wood nematode-carrying insects have been identified, with *Monochamus carolinensis* serving as the primary vector [7]. *M. alternatus*, also known as *Monochamus* (Coleoptera: Cerambycidae: Lamiinae: *Monochamus*), is found throughout most of China [8]. *M. alternatus* (Coleoptera: Cerambycidae: Lamiinae) is a significant stem borer of pine trees and serves as the primary vector of the pine wood nematode (*Bursaphelenchus xylophilus*), a major forest pest responsible for pine wilt disease. The species is widely distributed across East Asia, including China, South Korea, Japan, Laos, and Vietnam. In China, *M. alternatus* is particularly damaging, occurring across most provinces. In recent years, the species has expanded its range to higher latitudes, likely due to climate change and increased human-mediated transport [9]. The employment of quarantine and traceability techniques are effective in preventing and managing insect vectors, which helps manage pine wilt disease. The spread of insect vectors occurs in two ways: natural and artificial. Natural spread refers to the long-distance movement of insects through flight for activities such as mating, oviposition, and predation. Artificial spread, on the other hand, results from human activities, such as the transportation of insect larvae, eggs, or other forms during trade, transportation, and other economic activities. When invasive insects are detected in a new area, it is essential to trace their origin and implement quarantine and control measures promptly. Tracking the origin of insect vectors is crucial for predicting, managing, and mitigating pest damage. Developing a new tool to trace the source of the insects is highly important. Various pattern recognition tools have been created and used in food origin traceability [10]. Molecular markers (such as the mitochondrial COI gene) typically reflect the long-term evolutionary lineage or genetic structure of a species, and their mutation rate is relatively slow. For tracking short-term, small-scale geographical migrations of individuals over a period of days, weeks, or thousands of meters (such as the supply chain of agricultural products from a specific village to a market), molecular markers often fail to provide sufficient temporal and spatial resolution. Successful geographical tracing relies on matching unknown samples with reference databases of known geographical populations. However, there are significant sampling gaps globally (such as in Central Asia and parts of Africa), and databases often favor economic species or model organisms. The lack of high-coverage, standardized background reference libraries can lead to misjudgments or inability to trace. Although molecular markers such as COI and 28S rDNA have been widely used to analyze genetic differentiation among insect populations, these approaches often fail to provide direct information on the geographical origin of individuals. Stable isotope analysis offers an alternative approach by reflecting environmental and climatic signals incorporated during larval development. However, studies applying isotope-based traceability to forestry pest insects remain limited. Therefore, this study aims to establish a carbon isotope database for *M. alternatus* populations across China and evaluate the feasibility of using δ^13^C combined with geostatistical modeling to trace their geographical origin While measuring chemical parameters like fatty acids or triglycerides may not reliably classify geographical origins, the analysis of stable isotope ratios and multi-element data has been proposed as the most effective analytical method for accurately determining geographical origins [11].

There are two systems of nuclear DNA and mitochondrial DNA in organisms. Organisms possess two distinct genetic systems: nuclear DNA and mitochondrial DNA. Nuclear DNA contains both coding and non-coding regions, with highly conserved sequences such as 18S rDNA and 28S rDNA commonly used for phylogenetic analysis at higher taxonomic levels. Mitochondrial DNA, due to its maternal inheritance, lack of recombination, and relatively high mutation rate, is widely used for population genetics and phylogeographic studies [12,13,14]. This method is also widely used in the study of genetic differentiation in geographical insect populations. At present, nuclear genes (18S rDNA, 28S rDNA) and mitochondrial protein-coding genes (COI, COII, Cytb) are mainly used in molecular system research, and other molecular markers are less commonly used [12,13,14].

In recent years, stable isotope analysis has been employed to determine the geographical origins of insects [15,16,17,18]. The isotopic composition of insect tissues is derived from their diet through feeding interactions. For herbivorous insects, the stable isotope ratios of their bodies reflect those of their host plants, with predictable trophic discrimination [19,20,21,22]. Studies have utilized the difference between C3 and C4 plants to investigate insect movement between various host plants. Additionally, strontium isotope analysis combined with trajectory methods has been used to elucidate the overseas migration of *Mythimna separata* to Japan [23]. The research on insect migration relies on large-scale differences in isotope stability [24]. The migration of *Danaus plexippus* was examined using deuterium (D) and carbon-13 (^13^C) as markers [25]. The δD values in the wings of *Danaus plexippus* were measured by cultivating three groups of their host plant, *Asclepias curassavica*, using three water sources with distinct δD values. This confirmed the hypothesis that δD in the wings reflects the δD gradient resulting from varying rainfall levels in their birth regions. Additionally, an increasing gradient of δ^13^C in the wings from south to north was observed, suggesting that approximately half of the overwintering *Danaus plexippus* in Mexico originated from grain and legume crop areas in the U.S. Midwest. For the first time, the geographical distribution of δD and δ^13^C was mapped using the Kriging interpolation method from geostatistics. Stable isotope analysis is a powerful tool for studying trophic levels and the flow of materials and energy within ecosystems [26]. From producers to consumers across all levels, various isotopes exhibit regular changes in tandem with the increase in trophic level. The identification of isotopes can be used to construct food webs and determine trophic levels. It is crucial to select the appropriate isotope for accurate analysis. For instance, δ^13^C changes by 0.5‰ to 1% per trophic level, δ^15^N increases by 3% per trophic level, while δ^34^S remains largely unchanged across trophic levels [27]. The combined application of multiple isotopes can effectively reduce experimental error [24]. This study primarily focuses on *M. alternatus*, whose main hosts are pine species such as *Pinus massoniana*, *Pinus sylvestris*, and *Pinus thunbergii*. Because *M. alternatus* larvae feed on a limited range of host trees (primarily *Pinus massoniana*, *P. sylvestris*, and *P. thunbergii*), the carbon isotope composition of the insect directly reflects that of its host plant. This trophic specialization reduces dietary variability, making δ^13^C a more reliable geographical tracer compared to polyphagous insect species. As it has a single host, selecting carbon isotopes as the research object ensures higher accuracy. However, the flight behavior and spatial distribution of insects within forests are difficult to predict. As a result, tracing specific populations requires large-scale sampling, which is both time-consuming and labor-intensive. To address this, we supplement data from unexplored areas by predicting the spatial isotope distribution trends. These predictions are made using the Kriging interpolation method, which incorporates geostatistical and correlation analyses [28,29].

The spatial data for insect vectors in this study were obtained through field sampling. A broader sampling range results in more comprehensive data and greater accuracy for subsequent research. However, due to constraints in time and manpower, large-scale nationwide sampling was not feasible. Consequently, a limited number of sampling points were chosen to interpolate the δ^13^C values of vector insects, while the environmental variables most strongly correlated with δ^13^C were selected for co-Kriging interpolation. Finally, the differences between various models were analyzed. The difference model derived from this study’s findings can effectively trace the geographical origin of pine wood nematode insect vectors and provide a theoretical basis for the prevention and control of pine wood disease.

## 2. Materials and Methods

### 2.1. Sample Collection

**Figure 1:**
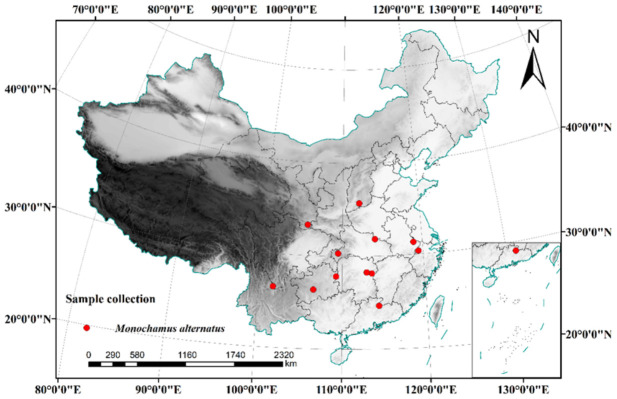
Sample collection coordinate points.

This study focused on areas affected by pine wilt disease and regions where *M. alternatus* is distributed (Figure 1). Samples were collected between 2019 and 2024 from 12 provinces across China: Sichuan (Panzhihua), Guizhou (Guiyang and Tongren), Hubei (Enshi), Jiangxi (Quannan), Zhejiang (Hangzhou), Gansu (Kangxian), Hunan (Changsha and Liuyang), Shanxi (Jincheng), Jiangsu (Nanjing), and Henan (Xingyang). A total of 850 adult specimens were captured using cross-plate traps (Model BF-10; Hangzhou Felomon Company, Hangzhou, China) baited with F8 attractant. Detailed information for each sampling site, including geographic coordinates, elevation, sampling date, and δ^13^C values, is provided in Table 1. The traps employed were *M. alternatus* cross-plate traps (Model BF-10), baited with an F8 attractant (Hangzhou Felomon Company, Hangzhou, China). Each trap was placed at least 4 km apart, with five traps set at each collection point. These traps were suspended in different forest structures and monitored every three days from June to August, and weekly from August to September. The captured insect samples were preserved individually in 5 mL centrifuge tubes filled with 99% ethanol, transported to the laboratory, and stored at −20 °C for further analysis.

### 2.2. Carbon Isotope Acquisition from M. alternatus Samples

*M. alternatus* specimens stored in the refrigerator were retrieved, and the abdominal cavity of each beetle was cut open using scissors and tweezers to remove the internal organs and undigested food, leaving only the exoskeleton for testing. Samples were first soaked in deionized water for 30 min, then immersed in a methanol–chloroform mixture (2:1, *v*/*v*) for 2 h for lipid removal. The solvent was then discarded. They were then washed twice with deionized water for 30 min each, soaked again in the methanol and chloroform mixture (2:1) for 2 h, and washed twice more with deionized water. The treated samples were then placed in a dry test tube and dried at 60 °C for 72 h. The dried samples were ground for 15 min and subsequently weighed using an analytical balance (accuracy of 0.001 g). The powdered samples were then wrapped in tin foil, left to stabilize at room temperature, and examined using a spectrometer. Isotope ratio measurements were conducted with the Isotope Ratio Mass Spectrometer (IRMS) at Tsinghua University (Shenzhen, Guangdong Province, China). Spatial data collected included latitude, longitude, altitude, and bioclimatic factors. The bioclimatic factor data were obtained from the WorldClim website (World Climate Database, 2020; Version 2.1, covering climate data from 1970 to 2000) (Bioclimatic variables—WorldClim 1 documentation, https://worldclim.org/data/bioclim.html, accessed on 20 March 2024) (Table 2).

In this study, 19 bioclimatic factors (Bio1-Bio19) were selected for analysis. These factors include Bio1: annual mean temperature; Bio2: mean diurnal range (mean of monthly (max temp–min temp)); Bio3: isothermality ((Bio2/Bio7) × 100); Bio4: temperature seasonality (standard deviation × 100); Bio5: maximum temperature of the warmest month; Bio6: minimum temperature of the coldest month; Bio7: annual temperature range (Bio5–Bio6); Bio8: mean temperature of the wettest quarter; Bio9: mean temperature of the driest quarter; Bio10: mean temperature of the warmest quarter; Bio11: mean temperature of the coldest quarter; Bio12: annual precipitation; Bio13: precipitation of the wettest month; Bio14: precipitation of the driest month; Bio15: precipitation seasonality (coefficient of variation); Bio16: precipitation of the wettest quarter; Bio17: precipitation of the driest quarter; Bio18: precipitation of the warmest quarter; and Bio19: precipitation of the coldest quarter.

Bioclimatic variables were derived from monthly temperature and rainfall data to produce variables that are more relevant to biological processes. These variables are commonly used in species distribution modeling and related ecological modeling techniques. They reflect annual patterns (e.g., mean annual temperature, total annual precipitation), seasonal changes (e.g., temperature and precipitation ranges throughout the year), and extreme or limiting environmental factors (e.g., the temperature of the coldest and warmest months, as well as precipitation during the wettest and driest quarters). Additionally, we included Bio20: national carbon dioxide concentration data and Bio21: atmospheric radiation data (https://data.cma.cn/data/cdcdetail/dataCode/G.0029.0001.S001.html, accessed on 20 March 2024). The δ^13^C values of the samples were determined using a Finnigan Delta V Advantage Isotope Ratio Mass Spectrometer (Thermo Fisher Scientific Inc., Waltham, MA, USA) and an EA-HT Elemental Analyzer (Thermo Fisher Scientific Inc., Waltham, MA, USA). The samples were first combusted at high temperatures in the elemental analyzer, generating CO_2_. Subsequently, the mass spectrometer measured the ^13^C/^12^C ratio of the CO_2_ and compared it to the international standard (Peedee Belemnite, PDB) to calculate the δ^13^C values. The accuracy of the determination was δ^13^C ± <0.1‰ (for unmarked samples) [18,30].

### 2.3. Data Processing and Analysis

Screening bioclimatic data: The 19 selected bioclimatic factors were categorized into two groups: temperature-related and precipitation-related factors. To minimize collinearity among these factors, the Pearson two-tailed test was applied to assess correlations between the data. Significant factors were then combined with carbon dioxide concentration and atmospheric radiation data, and the variance inflation factor (VIF) was used to evaluate the multicollinearity between them [31]. A VIF ≤ 10 indicates no collinearity among independent variables, while higher VIF values suggest more serious multicollinearity [32,33]. Linear regression analysis was conducted to examine the correlation between environmental variables, bioclimatic factors, and the carbon isotopes of insect vectors. The three climatic factors most strongly correlated with the sampling points were selected for co-Kriging interpolation modeling [34]. The closer the R^2^ value is to 1, the more reliable the model.

Before applying co-Kriging interpolation, we assessed the spatial autocorrelation of δ^13^C values using Moran‘s I statistic based on the sampling points. The result indicated significant positive spatial autocorrelation (Moran’s I = 0.47, *p* < 0.01), confirming that the δ^13^C values are spatially structured and that geostatistical interpolation is appropriate.

### 2.4. Model Validation and Progress Evaluation

The data from previous sampling points (latitude and longitude) and the carbon stable isotope data of *M. alternatus* were imported into ArcGIS 10.8. Consequently, co-Kriging interpolation was applied to the three selected bioclimatic factors and carbon isotope ratios, followed by the construction of 11 semi-variograms, and Kriging interpolation was used to establish candidate models. Cross-validation was then used to systematically screen these models, comparing them using four evaluation metrics: root mean square error, average standard error, root mean square standard error, and average standard error. Subsequently, exploratory spatial data analysis (ESDA) was conducted on models with similar evaluation indicators. Prognostic plots (scatter plots of predicted vs. measured values) and normal quantile-quantile plots (Q-Q plots) were generated for further analysis and comparison [35].

The leave-one-step cross-validation method was used to validate and evaluate the model within ArcGIS.

Step 1: The carbon isotope values of the samples were randomly divided into 65 samples without repetition.

Step 2: Each of the 65 samples was alternately selected as the validation sample, while the remaining 64 served as the training set.

Step 3: Step 2 was repeated 65 times, ensuring that each sample set had an equal chance of being selected as the test set. After training each set, a model was obtained, and it was tested on the corresponding test set. The evaluation index for each model was calculated and recorded.

Step 4: The results from 60 validations were averaged to produce the final result.

The model’s performance is considered good if the root mean square error and the average standard error are close to 0 or if the average standard error approaches the root mean square error. Additionally, the model is deemed effective if the root mean square standardization error is close to 1. However, if the root mean square standard error exceeds 1, it suggests that the variability in predictions is underestimated; if it is less than 1, the variability is overestimated.

## 3. Results

Based on the coordinates of our sample sampling points, we obtained data on 21 biological factors and will conduct further research based on these biological factors (Table 2).

### 3.1. Colinearity Test of Bioclimatic Variables

As detailed in Table 3, Bio9 (mean temperature in the driest quarter) showed the highest correlation with other factors in the two-tailed test of Bio1–Bio11 (Table 3), while Bio3 (isothermality) displayed no correlation with any of the factors. Consequently, Bio3 passed the collinearity test, and Bio9 (mean temperature in the driest quarter) was chosen as the representative factor with the highest correlation for further analysis. In the two-tailed test of Bio12–Bio19 (Table 4), Bio15 (precipitation seasonality) exhibited the lowest correlation, allowing it to pass the collinearity test. Bio12 (annual precipitation) and Bio17 (precipitation in the driest quarter) showed the highest correlation, leading to the selection of Bio15, Bio12, and Bio17 for subsequent analysis to assess their correlation with the carbon stable isotopes of insect vectors.

The variables Bio3, Bio9, Bio15, Bio12, Bio17, Bio20, and Bio21 were combined for a multicollinearity test. This test plays a very important role in multiple linear regression analysis, as it helps detect whether there is a high correlation between the independent variables in the model. This correlation can result in inaccurate estimates of regression coefficients, inflated standard errors, and unreliable model predictions. To assess the linear correlation between each pair of independent variables, the correlation coefficients were calculated. If there is a strong correlation among multiple independent variables, multicollinearity may be present. The VIF value is greater than 1, and the closer it is to 1, the weaker the multicollinearity. A VIF value of 10 is considered the threshold, and if VIF < 10, multicollinearity is absent. In the present case, the VIF values for annual precipitation, precipitation in the driest quarter, O_2_ concentration, and solar radiation were all less than 10, indicating no multicollinearity between them. However, the VIF values for isothermality, mean temperature in the driest quarter, and seasonality were between 10 and 20, suggesting mild collinearity among them (Table 5).

### 3.2. Correlation Analysis Between Carbon Stable Isotope Ratio of Insect Vectors and Environmental Climatic Factors

We examined the correlation between the carbon stable isotope ratio in insect vectors and environmental factors (latitude, longitude, altitude) [36]. The results indicate that the isotope ratios in insects are strongly correlated with environmental factors such as altitude and latitude [37,38]. Our analysis revealed significant correlations between the carbon stable isotope of insect vectors and two bioclimatic variables: Bio15 (precipitation seasonality) and Bio21 (solar radiation) at the 0.05 significance level (Table 6). Additionally, Bio12 (annual precipitation) and Bio17 (precipitation in the driest quarter) were also found to be significant, though their significance level was slightly lower compared to Bio15 and Bio21. Based on these findings, we selected these four factors as auxiliary variables and applied the co-Kriging interpolation method in ArcGIS to construct a spatial model.

**Figure 2:**
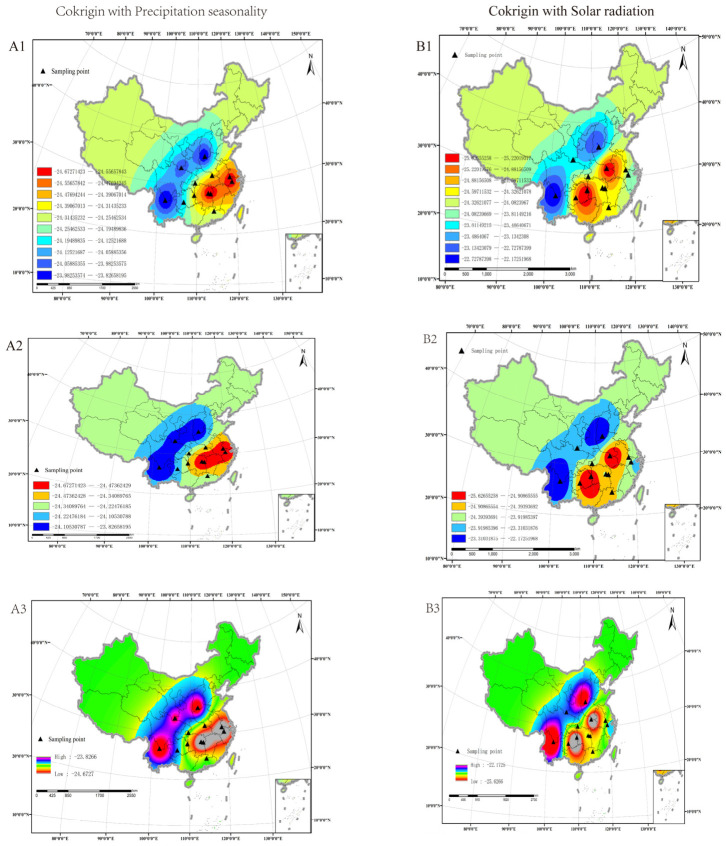
The spatial distribution of carbon isotope prediction values for *M. alternatus* in China. (**A1**–**A3**) are derived using co-Kriging interpolation based on Bio15 (precipitation seasonality), while (**B1**–**B3**) are interpolated using co-Kriging results from Bio21 (atmospheric radiation or solar radiation).

Based on the exponential semivariogram, we conducted co-Kriging interpolation on the carbon stable isotopes of M. alternatus samples, integrating precipitation seasonality and solar radiation data from 60 regions across 12 provinces in China. The results, presented in Figure 2, are color-coded, with values classified using the Jenks natural discontinuity classification method. Different classification types are denoted by (A1), (A2), (A3), and (B1), (B2), (B3). Theoretically, a greater number of classification groups results in more abundant levels, narrower isotope ratio intervals, smaller corresponding indication areas, and more precise results (e.g., (A1) and (B2)). Conversely, fewer classification groups result in larger isotope ratio intervals and less refined outcomes (e.g., (A2) and (B2)). In addition, the isotope ratio differences within the same collection region and between regions can influence grouping accuracy. In this study, except for the Jincheng area of Shanxi (where intra-group differences reached 0.21, *n* = 5), the intra-group differences in other regions ranged between 0.05 and 0.11 (*n* = 5). For six classification groups, the intra-group differences in carbon stable isotopes fell between 0.05 and 0.11 (*n* = 5). The overall difference was approximately 1. Despite the classification groups being less tightly grouped, the interpolation results remain reliable.

In addition to Pearson correlation analysis, we further evaluated the spatial cross-correlation between δ^13^C and each environmental variable using cross-semivariogram analysis. Bio15 (precipitation seasonality) and Bio21 (solar radiation) showed the strongest spatial coherence with δ^13^C, with clear spatial structure in their cross semivariograms. This supports their use as auxiliary variables in co-Kriging interpolation.

### 3.3. Model Construction and Verification

**Figure 3:**
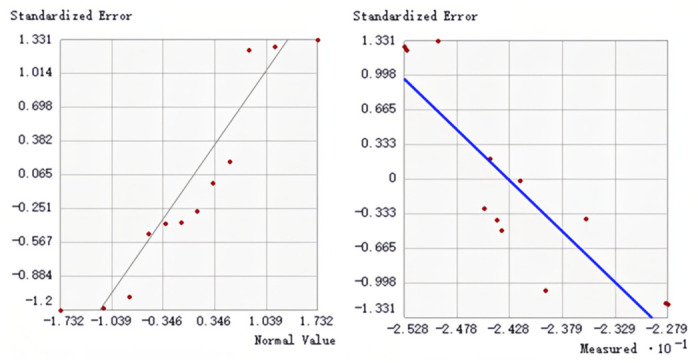
Model data analysis results (joint precipitation seasonality).

**Figure 4:**
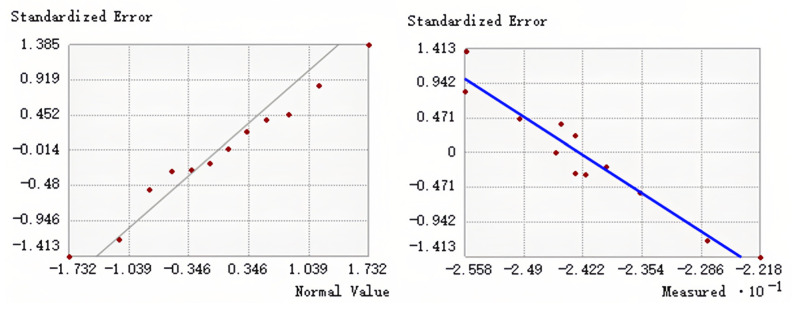
Model data analysis results (joint solar radiation).

Co-Kriging interpolation using different semivariogram models provides different results. Therefore, we use cross-validation to evaluate the accuracy of our model. Two models were obtained by co-Kriging interpolation of precipitation seasonality and solar radiation data with δ^13^ C values, respectively. The accuracy of 11 semivariogram candidate models for each type was tested and verified. The results are shown in Table 7 and Table 8. According to the cross-validation results in the table, the following indicators should be considered when evaluating each model: root mean square error (RMSE): the lower the better, reflecting the prediction accuracy. Mean standard error (Mean SE): the closer to 0, the better, indicating the prediction deviation. Key model comparison: J-Bssel: RMSE is the lowest (0.6300), and the prediction is the most accurate. RMSSE (0.9126) is the closest to 1, and the uncertainty estimation is reasonable. Mean SE (−0.1509) has a large negative deviation, which may be systematically underestimated. Hole effect: RMSE is the second lowest (0.6468), and the prediction accuracy is better. Mean SE (−0.0789) deviation is small, and the systematic error is relatively low. RMSSE (0.8917) is close to 1, but slightly inferior to J-Bssel. Based on the results of ESDA (Figure 3 and Figure 4), First, scatter plots of predicted versus measured values were examined to evaluate the degree of agreement with the 1:1 line. Secondly, we verify whether the red data points in the standard deviation normal Q-Q plot are evenly distributed along the gray line. If so, this indicates that the error between the predicted value and the measured value follows the normal distribution. Overall, the results showed that there was no significant difference in the prediction plots of the two semi-variogram models. The normal Q-Q diagram of the exponential model performs well, and two co-Kriging interpolation models are established based on this.

To select the optimal semivariogram model for co-Kriging, we compared 11 candidate models using cross-validation. The exponential model was selected for both co-Kriging interpolations (with Bio15 and Bio21) because it yielded the lowest root mean square error (RMSE) and the root mean square standardized error (RMSSE) closest to 1 (Table 7 and Table 8), indicating the best balance between prediction accuracy and reliable uncertainty estimation.

### 3.4. Spatial Distribution of Carbon Isotope Prediction Values of M. alternatus

**Figure 5:**
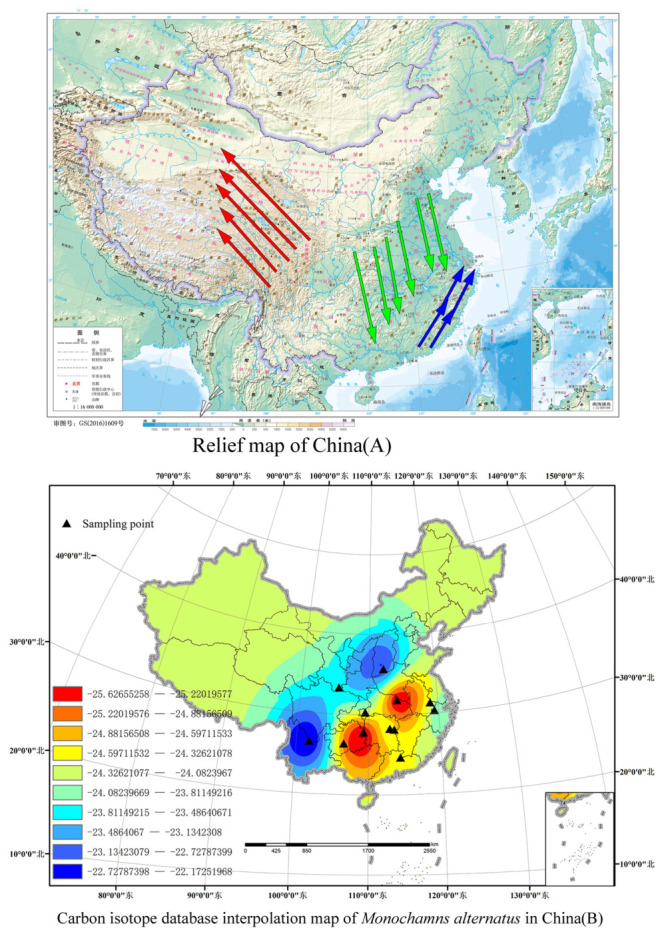
China topographic map and carbon isotope interpolation map of *Monochamus alternatus.* Red arrow: High-altitude areas, Green arrow: Plain area, Blue arrow: Hilly area.

Comparing our database results with China’s topography, it is found that the interpolation results of the carbon isotope database of *M. alternatus* tend to be consistent with China’s altitude trend. Our low-value area (−25.62–24.52) coincides with the Central China Plain area of China’s topography map (the area covered by green arrows in Figure 5A), the median area (−23.994–24.32) coincides with the Jiangnan hilly area (the area covered by blue arrows in Figure 5A), and the high-value area (−23.99–22.17), which diffuses with the increase in altitude (area covered by the red arrows in Figure 5A).

The results derived from co-Kriging interpolation incorporating precipitation seasonality and solar radiation are essentially the same. The interpolation outcomes can be divided into three regions based on carbon isotope levels: low, medium, and high. Specifically, the low-value region ranges from −22 to −23, the medium-value region from −23 to −24, and the high-value region from −24 to −25. In Figure 2(A1,A2), the high-value region is marked in blue, the medium-value region in orange, and the low-value region in blue. In order to distinguish the two co-Kriging interpolation regions, Figure 2(B1) provides a clearer visualization. In Figure 2(B2), the high-value region is represented in red; from the isotope variation in the figure, it is evident that the carbon isotope values of *M. alternatus* display a gradient change. The carbon isotope changes in *M. alternatus* exhibit a trend from high to low from China’s Qinling Mountains–Huaihe River Line to the east. As the carbon isotope values of *M. alternatus* approach the threshold of the adjacent interval, it can be inferred that the origin of the species is likely near the geographical boundary.

### 3.5. Carbon Isotope Database Verification of M. alternatus

After the establishment of the carbon isotope database of *M. alternatus*, we extracted the carbon isotope of the newly collected samples of *M. alternatus* from Ningshan County, Shaanxi Province, Chengdu City, Sichuan Province, and Weihai City, Shandong Province, and then substituted the carbon isotope of the samples into the carbon isotope database. It was found that the carbon isotope value is accurately indicated in the database (Table 9).

**Figure 6:**
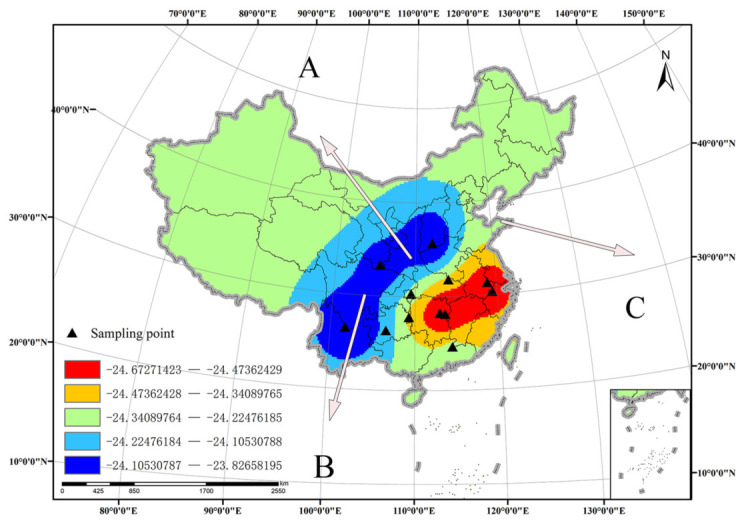
Carbon isotope database validation.

By replacing the carbon isotope values of Ningshan County in Shaanxi Province, Chengdu in Sichuan Province and Weihai in Shandong Province into the Monochamus alternatus carbon isotope database, we found that the samples from Shaanxi Province were in the high-value region (Figure 6(A)), while the samples from Sichuan and Shandong were in the middle-value region (Figure 6(B,C)). This result, combined with the hydrogen isotope and local precipitation isotope, allows us to accurately trace the geographical source of Monochamus alternatus, facilitating effective management and prevention of pine wood nematode disease.

## 4. Discussion

In this study, we established a carbon isotope database for *M. alternatus* in China to enhance efforts in controlling pine wilt disease, prevent the spread of the insect vector *M. alternatus*, and trace its origins. Utilizing samples of *M. alternatus* collected from 12 provinces across China, we analyzed their carbon isotope data. In order to identify the optimal mathematical model, we tested and examined various environmental factors, ultimately selecting the two most strongly correlated variables: precipitation seasonality and solar radiation. Using this mathematical model, we conducted co-Kriging interpolation in ArcGIS to generate a carbon stable isotope interpolation map for *M. alternatus* in China. The interpolation results derived from both precipitation seasonality and solar radiation were found to be largely consistent.

The results showed that precipitation seasonality (Bio15) and solar radiation (Bio21) were the most important factors influencing the carbon isotope ratio of *M. alternatus*. These findings can be explained by the underlying physiological mechanisms at the plant level and the subsequent transfer of isotopic signals through the food chain.

At the plant level, carbon isotope discrimination during photosynthesis is primarily controlled by stomatal conductance and carboxylation efficiency. Precipitation seasonality (Bio15) reflects the temporal distribution of water availability, which directly affects plant water status. Under conditions of water limitation or seasonal drought, pine trees close their stomata to reduce transpirational water loss. This stomatal closure reduces the diffusion gradient of CO_2_ into the leaf, leading to decreased discrimination against ^13^CO_2_ and resulting in less negative (more enriched) δ^13^C values in plant tissues. Conversely, periods of adequate water availability allow for greater stomatal opening, increasing discrimination and yielding more negative δ^13^C values. Solar radiation (Bio21), on the other hand, influences photosynthetic photon flux density and the rate of carbon fixation. Higher solar radiation can enhance photosynthetic capacity but may also induce photoinhibition or water stress, indirectly affecting stomatal behavior and isotopic fractionation.

The isotopic signal established in host plants is then transmitted to *M. alternatus* through the food chain. As a monophagous or oligophagous herbivore, *M. alternatus* larvae feed exclusively on the phloem and xylem of pine trees during their developmental period. The carbon isotope composition of insect tissues reflects that of their diet, with minimal trophic discrimination typically ranging from 0.5‰ to 1‰. In this study, we used the adult exoskeleton for analysis, which is metabolically inert after eclosion and preserves the isotopic signature accumulated during larval feeding. Therefore, the δ^13^C values of *M. alternatus* serve as a reliable integrator of the environmental conditions experienced by its host trees during the growing season.

The combined influence of precipitation seasonality and solar radiation on plant δ^13^C, coupled with the direct trophic transfer of this signal to herbivorous insects, explains why these two environmental variables emerged as the primary drivers of spatial variation in *M. alternatus* δ^13^C. This mechanistic understanding not only supports the use of carbon stable isotopes for geographical tracing but also highlights the potential of insect isotopic composition as a proxy for environmental conditions in forest ecosystems.

Our sampling data currently covers only 12 provinces in China, with just five sampling points in each province. The distribution of *M. alternatus* in China spans most regions except for the northeast, as well as Gansu, Qinghai, and Xinjiang. To create a more comprehensive carbon stable isotope database for *M. alternatus*, it is necessary to expand the sampling area and include more regional data. Boundary delimitation is a critical issue; in fact, abrupt changes in isotope values are expected near boundaries, depending on the analyzed sampling points. As the number of sampling points increases, the boundary transition becomes smoother. In practical applications, when the δ^13^C value of *M. alternatus* is near the critical value, its distribution tends to align with the boundary. Since we average the data during analysis, it reflects the center of the region. This situation can be addressed by increasing the number of samples for analysis. The sampling points chosen for this study were selected to ensure better coverage of provinces where pine wilt disease has occurred and is more widespread. However, sampling was not conducted in different regions within the same province. Radial line sampling, a statistical method commonly used in forest science and other fields, can be employed for this purpose, especially when estimating coverage in small, irregularly shaped areas. This technique involves starting at a fixed point in the region and sampling along a randomly chosen direction (azimuth). By using this approach, the quantitative aspects of the spatial structure can be assessed, resembling the local stereology technique. This method helps reduce variance in the estimation process, thereby improving the accuracy and reliability of the estimation [39]. Although pine wilt disease has affected some provinces that were not sampled, future research by our group will involve large-scale sampling in these areas to further enrich the Chinese *M. alternatus* carbon isotope database.

While there are various traceability technologies, each comes with its own set of advantages and disadvantages, and no single technology can be fully applied to origin traceability. Therefore, to enhance the accuracy of traceability identification, it is essential to combine different technologies and analyze various substances in the product, while also selecting the appropriate chemometrics for different test results. This approach applies to both food and insect traceability. The key to effective traceability lies in creating a reliable database for discriminant analysis. Currently, molecular traceability has a relatively comprehensive database and available data. In contrast, stable isotope traceability lacks a unified database and an established, complete traceability system, which has resulted in limited comparison across different research studies to date. At present, research on food traceability is abundant and varied. Srayko et al. employed stable isotopes, such as S, to investigate the seasonal migration of aquatic insects (Hemiptera: *Nymphalidae*), highlighting a significant transfer of resources between wetland and river ecosystems [40]. However, studies focused on insect traceability are limited. In previous studies, someone has studied hydrogen isotopes to explore the geographical origin of *Lymantria dispar* L. [41], with the hope of encouraging further research on insects and other agricultural and forestry pests in the future.

*Monochamus alternatus* is not only the main vector insect of pine wilt disease in China, but also the main vector insect of pine wilt disease in Japan and South Korea in Asia, which has caused serious damage to pine forests in Japan and South Korea [42,43]. *Monochamus alternatus* is not only the main vector insect of pine wood nematode disease in China, but also the main vector insect of pine wood nematode disease in Asia, causing serious harm to pine forests in Japan and South Korea. Northeast China is close to South Korea and has a lot of trade with Japan and South Korea. If the larvae or adults of *Monochamus alternatus* are found in wood packaging, we can use this carbon isotope database to verify whether the sample is domestic. If not, it is most likely to be spread by neighboring countries during trade. In future quarantines, testing can be strengthened to provide data support for the prevention, control and detection of pine wood nematode disease.

## 5. Conclusions

In the present study, the carbon isotope database for *M. alternatus* in China was developed, and by incorporating co-Kriging interpolation with environmental parameters, the spatial distribution of its carbon stable isotopes was effectively predicted. The findings reveal that the seasonality of precipitation and solar radiation are the key environmental factors influencing the carbon isotopes of *M. alternatus*. Additionally, the carbon isotope distribution exhibits a west-to-east gradient, decreasing from high to low. This study provides a scientific foundation for the prevention and control of pine wilt disease and for tracing the geographic origins of its insect vectors.

However, several limitations should be acknowledged when interpreting these findings. First, the geographic coverage of sampling is currently limited to 12 provinces in China. Although these regions represent the major areas affected by pine wilt disease, the absence of samples from northeastern China, western provinces (such as Qinghai and Xinjiang), and neighboring countries (including Japan, South Korea, and Vietnam) restricts the applicability of the current database for tracing transboundary introductions or long-distance dispersal events. Second, this study relies on a single isotope (δ^13^C). While carbon isotopes effectively capture variation in water availability and photosynthetic conditions, they may not fully resolve geographic origins in regions with overlapping δ^13^C signatures. The integration of additional isotopes—particularly δ^2^H and δ^18^O, which reflect precipitation patterns, and δ^15^N, which captures anthropogenic and edaphic influences—would substantially enhance discrimination power and should be prioritized in future research. Third, host plant effects represent a potential source of variability not fully accounted for in this study. *M. alternatus* larvae feed on multiple *Pinus* species, including *P. massoniana*, *P. sylvestris*, and *P. thunbergii*, each of which may exhibit species-specific δ^13^C baselines due to differences in photosynthetic physiology, phenology, and microhabitat preferences. Although our sampling sites were predominantly in *P. massoniana*-dominated forests, variation in host plant composition across regions may introduce additional isotopic variability. Fourth, with only five individuals per sampling site, intra-population variability may not be fully captured, and the relatively sparse sampling density may affect the precision of spatial interpolation, particularly in topographically complex regions.

Despite these limitations, the current study provides a foundational isoscape for *M. alternatus* in China and demonstrates the utility of carbon stable isotopes combined with spatial modeling for insect geographical tracing. Future research should focus on (1) expanding sampling coverage to un-sampled regions within China and neighboring countries, (2) incorporating multiple stable isotopes (δ^2^H, δ^18^O, δ^15^N) to improve discrimination power, (3) systematically recording host plant species to account for potential host-driven isotopic variation, and (4) increasing sample density to enhance the accuracy and resolution of spatial interpolation models. These efforts will collectively advance the development of a robust, multi-isotope traceability system for the management of pine wilt disease.

## Figures and Tables

**Table 1:** Sample collection information and geographic information.

Sample	Place	Latitude	Longitude	Elevation (m)	Carbon Isotope Ratios (%)	Sampling Time	Host	Collection Quantity
*Monochamus alternatus* (*n* = 5)	Sichuan (Panzhihua)	101.7381	26.4984	1800	−81 ± 0.02	2020.7	*Pinus massoniana* Lamb	52
*Monochamus alternatus* (*n* = 5)	Guizhou (Guiyang)	106.5517	26.3713	1077	−73 ± 0.16	2020.8	*Pinus massoniana* Lamb	90
*Monochamus alternatus* (*n* = 5)	Guizhou (Tongren)	109.2643	27.8162	2000	−74 ± 0.11	2020.8	*Pinus massoniana* Lamb	80
*Monochamus alternatus* (*n* = 5)	Hubei (Enshi)	109.4865	30.3010	1200	−68 ± 0.05	2020.7	*Pinus massoniana* Lamb	90
*Monochamus alternatus* (*n* = 5)	Jiangxi (Quannan)	114.3896	24.6530	329	−55 ± 0.05	2019.9	*Pinus massoniana* Lamb	66
*Monochamus alternatus* (*n* = 5)	Zhejiang (Hangzhou)	119.4364	30.3537	1500	−67 ± 0.21	2021.7	*Pinus massoniana* Lamb	40
*Monochamus alternatus* (*n* = 5)	Gansu (Kangxian)	105.5389	33.2978	1900	−66 ± 0.07	2023.8	*Pinus armandii* Franch	88
*Monochamus alternatus* (*n* = 5)	Hunan (Changsha)	112.9862	28.2559	1500	−54 ± 0.07	2022.9	*Pinus massoniana* Lamb	60
*Monochamus alternatus* (*n* = 5)	Shanxi (Jincheng)	112.1724	35.6873	814	−66 ± 0.06	2022.6	*Pinus massoniana* Lamb	60
*Monochamus alternatus* (*n* = 5)	Jiangsu (Nanjing)	118.8921	31.3275	100	−52 ± 0.16	2023.7	*Pinus massoniana* Lamb	68
*Monochamus alternatus* (*n* = 5)	Henan (Xingyang)	114.0953	31.8118	143	−51 ± 0.11	2022.7	*Pinus massoniana* Lamb	90
*Monochamus alternatus* (*n* = 5)	Hunan (Liuyang)	113.6122	28.1451	1150	−50 ± 0.06	2021.8	*Pinus massoniana* Lamb	66

**Table 2:** The longitude, latitude, altitude, and biological variables of the sampling sites and δ13C values of the insect vectors.

Site	Lon	Lat	Elo	δ^13^C	Bio1	Bio2	Bio3	Bio4	Bio5	Bio6	Bio7	Bio8	Bio9	Bio10	Bio11	Bio12	Bio13	Bio14	Bio15	Bio16	Bio17	Bio18	Bio19	BIio20	Bio21
KX	105.54	33.30	1900.00	−23.56 ± 0.02	11.14	8.68	29.39	761.01	25.56	−3.96	29.53	19.31	1.36	20.30	1.36	686.00	134.00	3.00	82.31	362.00	14.00	340.00	14.00	409.51	5127.62
LY	113.61	28.15	1150.00	−24.53 ± 0.10	17.27	7.77	24.90	853.72	32.90	1.69	31.21	21.51	8.41	27.47	6.50	1468.00	219.00	46.00	49.03	638.00	184.00	452.00	204.00	406.26	3678.12
TR	109.26	27.82	2000.00	−25.28 ± 0.11	16.14	8.19	27.56	793.99	30.94	1.22	29.72	20.23	6.09	25.62	6.09	1261.00	210.00	30.00	58.04	558.00	107.00	478.00	107.00	409.79	2830.01
HZ	119.44	30.35	1500.00	−23.95 ± 0.05	11.74	7.27	24.45	820.71	26.24	−3.48	29.72	19.49	3.43	21.60	1.38	1550.00	241.00	40.00	49.38	608.00	156.00	607.00	173.00	407.09	4693.32
XY	114.10	31.81	143.00	−25.26 ± 0.05	13.44	8.20	26.00	868.19	28.50	−3.04	31.54	23.87	2.40	23.87	2.40	1182.00	213.00	20.00	59.29	521.00	93.00	521.00	93.00	411.25	5355.72
JC	112.17	35.69	814.00	−22.79 ± 0.21	10.38	11.10	30.18	967.53	27.72	−9.07	36.79	20.46	−2.11	21.86	−2.11	575.00	140.00	4.00	92.37	340.00	16.00	324.00	16.00	404.78	5665.75
ES	109.49	30.30	1200.00	−24.19 ± 0.07	16.49	7.74	25.99	796.84	31.85	2.08	29.77	23.99	6.38	26.04	6.38	1467.00	244.00	26.00	63.11	654.00	91.00	637.00	91.00	407.28	3931.23
PZH	101.74	26.50	1800.00	−22.81 ± 0.07	19.96	11.78	46.64	480.47	30.74	5.49	25.25	24.92	13.58	24.93	13.58	766.00	168.00	6.00	94.15	454.00	21.00	365.00	21.00	413.52	8864.60
QN	114.39	24.65	329.00	−24.47 ± 0.06	19.19	8.64	32.14	661.90	31.65	4.76	26.89	22.86	12.09	26.68	10.54	1726.00	306.00	37.00	63.03	810.00	139.00	622.00	193.00	412.45	6131.11
NJ	118.89	31.33	100.00	−24.31 ± 0.16	16.08	7.45	23.35	904.14	31.63	−0.28	31.92	24.56	6.83	26.91	4.64	1157.00	183.00	30.00	49.88	473.00	125.00	471.00	128.00	412.80	5538.57

**Table 3:** Results of the correlation analysis for Bio1–Bio11.

	Bio1	Bio2	Bio3	Bio4	Bio5	Bio6	Bio7	Bio8	Bio9	Bio10	Bio11
Bio1	1	−0.68 *	0.28	−0.87 **	0.78 **	0.97 **	−0.89 **	0.77 **	0.98 **	0.88 **	0.98 **
Bio2	−0.68 *	1	0.43	0.54 *	−0.46	−0.73	0.72 **	−0.38	−0.67 **	−0.65	−0.64 *
Bio3	0.28	0.43	1	−0.51	−0.03	0.24	−0.30	0.30	0.30	0.38	−0.06
Bio4	−0.87 **	0.54 *	−0.51	1	−0.40	−0.92 **	0.97 **	−0.62	−0.92 **	−0.55 *	−0.94
Bio5	0.78 **	−0.46	−0.03	−0.40	1	0.66 *	−0.44	0.67 **	0.69 **	0.96 **	0.66 **
Bio6	0.97 **	−0.73	0.24	−0.92 **	0.66 *	1	−0.96 **	0.71 **	0.98 **	0.80 **	0.99 **
Bio7	−0.89 **	0.72 **	−0.30	0.97 **	−0.44	−0.96 **	1	−0.61 *	−0.93 **	−0.62 *	−0.95 **
Bio8	0.77 **	−0.38	0.30	−0.62	0.67 **	0.71 **	−0.61 *	1	0.73 **	0.72 **	0.74 **
Bio9	0.98 **	−0.67 **	0.30	−0.92 **	0.69 **	0.98 **	−0.93 **	0.73 **	1	0.82 **	0.99 **
Bio10	0.88 **	−0.65	0.38	−0.55 *	0.96 **	0.80 **	−0.62 *	0.72 **	0.82 **	1	0.79 **
Bio11	0.98 **	−0.64 *	−0.06	−0.94	0.66 **	0.99 **	−0.95 **	0.74 **	0.99 **	0.79 **	1

* indicates that the correlation was significant at the 0.05 level (2-tailed). ** indicates that the correlation was significant at the 0.01 level (2-tailed).

**Table 4:** Results of the correlation analysis for Bio12–Bio19.

	Bio12	Bio13	Bio14	Bio15	Bio16	Bio17	Bio18	Bio19
Bio12	1	0.92 *	0.89 **	−0.85 **	0.76 **	0.86 **	−0.84 **	0.88 **
Bio12	0.89 **	1	0.70 **	0.70 **	0.70 **	−0.69 **	0.91 **	0.91 **
Bio14	−0.85 **	0.70 **	1	−0.89	0.79 **	0.69 **	0.99 **	0.98 **
Bio15	−0.87 **	0.70 **	−0.89	1	−0.68	−0.90 **	−0.60 *	−0.85
Bio16	0.76 **	0.70 **	0.79 **	−0.68	1	0.74 **	0.83 **	0.80 **
Bio17	0.86 **	−0.69 **	0.69 **	−0.90 **	0.74 **	1	0.52	0.98
Bio18	−0.84 **	0.91 **	0.99 **	−0.60 *	0.83 **	−0.96 **	1	0.55 *
Bio19	0.88 **	0.91 **	−0.62	−0.85	0.80 **	0.71 **	0.55 **	1

* the same as Table 3; ** the same as Table 3.

**Table 5:** Multicollinearity test.

Bioclimatic Variable	Collinearity Statistics VIF
Isothermality	16.366
Mean temperature in the driest quarter	14.929
Precipitation seasonality	28.495
Annual precipitation	5.478
Precipitation in the driest quarter	7.784
CO_2_ concentration	1.316
Solar radiation	2.806

**Table 6:** Correlation analysis of the stable carbon isotope ratio of vector insects with environmental factors and bioclimatic variables.

Environmental Factor	Pearson’s r	R^2^	Significance
Lon	−0.21454	−0.04070	-
Lat	0.20306	−0.04593	-
Elo	0.22437	−0.03599	-
Isothermality	0.51498	0.19841	-
Mean temperature in the driest quarter	−0.07579	−0.08464	-
Precipitation seasonality	0.56220	0.25390	**
Annual precipitation	−0.54733	0.23590	-
Precipitation in the driest quarter	−0.50062	0.18250	-
CO_2_ concentration	−0.28293	−0.00358	-
Solar radiation	0.60356	0.30649	**

Note: Lon: longitude; Lat: latitude; Elo: altitude. **: *p* < 0.01.

**Table 7:** Cross-validation results for the candidate models (joint precipitation seasonality).

Model Types	Root Mean Square Error	Mean Standard Error	Root Mean Square Standardized Error	Average Standard Error
circular	0.6804	−0.0498	0.8380	0.8105
Spherical	0.6725	−0.0429	0.8440	0.7959
Pentaspherical	0.6669	−0.0228	0.8696	0.7667
Tetraspherical	0.6690	−0.0359	0.8557	0.7813
exponential	0.6797	−0.0293	0.8251	0.8233
Gaussian	0.6939	−0.0607	0.8457	0.8184
rational quadratic	0.6794	−0.0272	0.8297	0.8182
Hole effect	0.6468	−0.0789	0.8917	0.7243
K-Bssel	0.6824	−0.0348	0.8305	0.8208
J-Bssel	0.6300	−0.1509	0.9126	0.6892
Stable	0.6812	−0.0324	0.8277	0.8223

**Table 8:** Cross-validation results for the candidate models (joint solar radiation).

Model Types	Root Mean Square Error	Mean Standard Error	Root Mean Square Standardized Error	Average Standard Error
circular	0.9016	−0.0910	1.1447	0.7438
Spherical	0.8593	−0.0670	1.0385	0.7791
Pentaspherical	0.8835	0.06349	1.3217	0.64089
Tetraspherical	0.9134	0.0813	1.3961	0.6316
exponential	0.8408	0.0526	1.1474	0.7095
Gaussian	0.9800	−0.1397	1.3096	0.7284
rational quadratic	0.7894	−0.0440	0.9269	0.7932
Hole effect	0.8982	−0.0731	1.3703	0.6472
K-Bssel	0.8567	−0.054	1.1255	0.7349
J-Bssel	0.7881	−0.1552	1.1951	0.6539
Stable	0.8835	−0.0636	1.1334	0.7380

**Table 9:** Verify the sampling information of the data.

Sample	Sampling Location	Latitude	Longitude	Elevation (m)	δ^13^C
*Monochamus alternatus* (*n* = 5)	Shaanxi (Ningshan)	108.1420	33.3484	1127	−23.4428
*Monochamus alternatus* (*n* = 5)	Sichuan (Chengdu)	103.6889	30.6156	380	−24.1002
*Monochamus alternatus* (*n* = 5)	Shandong (Weihai)	122.1005	36.5006	22	−24.1352

## Data Availability

The original contributions presented in this study are included in the article. Further inquiries can be directed to the corresponding author.
